# Regulation of protein flexibility and promoting the formation of high internal phase emulsions in peanut protein isolate using dual succinylation and ultrasonication modifications

**DOI:** 10.1016/j.fochx.2025.102727

**Published:** 2025-07-03

**Authors:** Fuwei Sun, Genna Ba, Chaojiang Dong, Zhijun Fan, Hongjian Zhang, Lulu Shen, Lianzhou Jiang, Zhongjiang Wang, Zengwang Guo

**Affiliations:** a College of Food Science, Northeast Agricultural University, Harbin, Heilongjiang 150030, China; b Inner Mongolia Yili Industrial Group, Co.Ltd., Hohhot, Inner Mongolia 010110, China; c Heilongjiang Beidahuang Green and Healthy Food Co., Ltd., Jiamusi, Heilongjiang 154007, China; d Hainan Institute of Grain and Oil Science, Qionghai, Hainan 571400, China

**Keywords:** Peanut protein isolate, Succinylation, Ultrasonication, Dual modifications, High internal phase emulsions

## Abstract

In order to obtain a high self-supporting capacity protein-based high internal phase emulsions (HIPEs) suitable for food 3D printing, the peanut protein isolate (PPI)-HIPEs was prepared by improving the protein flexibility under dual succinylation and ultrasonication modification. It has been found that the succinylated PPI (PPI-DS) showed decreased particle size, ζ-potential, and surface hydrophobicity, but increased protein flexibility and solubility. Following the succinylation modification, ultrasound induced additional improvements in these characteristics. Of these, the PPI-DS-300 W had the highest performance. The combination of dual succinylation and ultrasonication significantly altered the secondary structure of PPI, disrupting rigid globular structures and enhancing molecular flexibility. The improvement of protein flexibility decreased interfacial tension and strengthened interactions at the protein-oil interface, which further optimized its emulsifying performance. Furthermore, the dual succinylation and ultrasonication modifications generated stronger gel-like network structures to enhance the mechanical strength and deformation resistance of the HIPEs.

## Highlights

• Succinylation increased conformational flexibility of PPI.
• Ultrasonication enhanced the interfacial and emulsifying properties of PPI-DS.
• PPI-DS-300 W promoted the formation of HIPEs with stronger gel network.
• Dual modification improved printing performance of HIPEs.

## Graphical abstract

**Figure:**
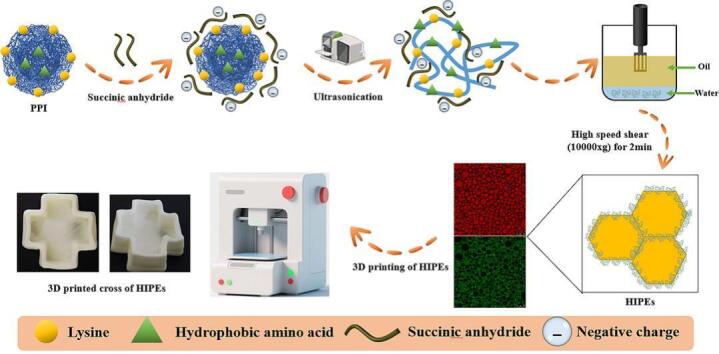

## 1 Introduction

High internal phase emulsions (HIPEs) are concentrated emulsions characterized by an oil volume fraction greater than 0.74, giving them a semi-solid appearance and the ability to support themselves (Hong, Kong, Guo, & Huang, 2024). In recent years, HIPEs have been extensively applied in industrial fields such as fat substitutes, 3D printing, and delivery of hydrophobic nutrients due to their exceptional properties (Dai et al., 2024; He & Lu, 2024; Zhang, Wang, Jiang, Min, & Rao, 2023). As natural biopolymers with amphiphilic and interfacial active properties, plant proteins are crucial in forming and stabilizing HIPEs by adsorbing at the oil-water interface to create dense interfacial films (Ji, Wang, Ma, Liang, & Luo, 2025). Consequently, plant proteins have increasingly been favored as emulsifiers for stabilizing HIPEs. Peanut protein isolate (PPI), sourced mainly from the by-product of peanut oil extraction, represents an underutilized resource with significant potential for valorization. Compared to widely studied plant proteins (e.g., soy, pea), PPI offers economic advantages due to its abundance in peanut-producing regions (e.g., China, India) and aligns with circular bioeconomy goals. However, native PPI suffers from poor solubility and emulsification capacity due to its rigid globular structure, limiting its application in HIPEs (Chen et al., 2025). Therefore, utilizing native PPI as a stabilizer for HIPEs remains a formidable task.

The spatial structure of protein molecules could be easily modified in regions referred to as flexible domains, whereas structures with limited modification capacity are termed rigid domains (Zhu et al., 2020). Generally, proteins with greater flexibility often exhibit enhanced interfacial and emulsifying properties (Yan, Xu, Zhang, & Li, 2021). Greater protein flexibility allows for better adsorption and rearrangement at the oil-water interface, which strengthens the viscoelasticity of the interfacial network and enhances steric hindrance between droplets (Lian et al., 2023). Chemical, physical, and biological strategies can be employed to adjust protein flexibility, thereby improving emulsifying performance. Among these, Succinylation influences protein structural flexibility, surface charge density, and functional properties through the interaction of succinic anhydride (SA) with amino acid residues like lysine, hydroxyl, and sulfhydryl groups (Lian et al., 2023). Furthermore, the SA offers advantages such as low cost, safety, non-toxicity, high efficiency, and controllability. Recent studies have demonstrated that succinylation-induced interactions with proteins can dissociate protein subunits, enhance flexibility, and promote structural unfolding and rearrangement at interfaces, thereby improving emulsion stability. Examples include soy protein (Lian et al., 2023), chickpea protein (Patil et al., 2024), egg white protein (Wu et al., 2022), myofibrillar protein (Lang et al., 2024), and antarctic krill protein (Wang et al., 2022). Additionally, physical processing methods, such as ultrasound treatment, can alter protein conformation and structure via cavitation, improving protein functional characteristics (Diao et al., 2024). Ultrasound typically preserves the primary structure of proteins, keeping them intact, but significantly alters their secondary and tertiary structures. For example, Wu et al. (2024) found that ultrasound treatment promoted the unfolding of tertiary structures, loosening molecular arrangements, increasing flexibility, and enhancing both interfacial viscoelasticity and emulsion stability. Cui et al. (2020) reported that ultrasound disrupted rigid domain structures and increased protein flexibility, facilitating adsorption and unfolding at the oil-water interface, ultimately improving the emulsifying properties of soy protein.

Despite the significant research on protein modification methods in recent years, single modification methods still exhibit certain limitations, failing to ensure optimal protein functionality (Zhang et al., 2023). Dual modification refers to the combination of two distinct sequential or repeated single approaches (e.g., dual physical/chemical/biological, physicochemical, or physical-biological methods) (Kamani, Semwal, & Khaneghah, 2022). Compared to single modification, dual modification enhances protein hydration, rigid/flexible domain distribution, and emulsifying properties in a single step. Wang et al. (2024) reported that the synergistic integration of succinylation with glycosylation significantly improved the functional properties of walnut glutenin. The combined succinylation-glycosylation approach enhanced protein rheological and emulsifying characteristics by promoting structural unfolding, reducing particle size, and increasing flexibility, outperforming individual modifications. Similarly, the combined enzymatic and ultrasonic modification increased surface properties and structural unfolding of protein, promoting the creation of a compact gel network with high strength (Wu et al., 2024). However, while some studies have explored the effects of dual modification on structure and emulsification performance of plant proteins (e.g., soy, pea), strategies to enhance PPI functionality, particularly through synergistic physical-chemical approaches, remain underexplored, and the research on how dual modification regulates the structural flexibility and interfacial behavior of PPI remains limited. Additionally, the effect of protein structural flexibility on the rheological characteristics of HIPEs and the ability of HIPEs to be 3D printed is still not well understood. These significantly limit the use of peanut protein as an emulsifier in the food sector.

Therefore, this research examined how dual succinylation and ultrasonication modifications impact the structure and functionality of PPI, as well as its stabilization mechanisms for HIPEs. First, structural and surface hydrophobic properties were characterized to analyze conformational changes in PPI induced by dual modification. Subsequently, the relationship between protein flexibility, secondary structure content, and interfacial/emulsifying behaviors was systematically investigated. Finally, HIPEs stabilized by PPI were successfully prepared, and their applicability in food 3D printing was confirmed by analyzing the changes in microstructure, rheological properties, and self-supporting capacity of HIPEs stabilized with different modified PPI samples.

## 2 Materials and methods

### 2.1 Materials

The peanut protein isolate (PPI, 91.21 % of proteins), succinic anhydride (SA, ≥99.0 %), ninhydrin (≥98.0 %) and porcine pancreatic trypsin (specific activity 2500 units/mg), were purchased from Yuanye Bio-Technology Co., Ltd. (Shanghai, China). Food-grade soybean oil was commercially acquired from Jiusan Grain and Oil Industry Group Co., Ltd. (Harbin, China). All remaining chemicals consisted of analytical-grade reagents routinely used in laboratory protocols.

### 2.2 Treatment of PPI through succinylation and ultrasonication

PPI succinylation was performed based on the protocol by Wan, Liu, and Guo (2018) with minor modifications. A 20 mg/mL PPI solution was prepared by stirring PPI powder in deionized water for 3 h at 25 °C. The pH was then brought to 8.0 with the addition of 2 mol/L NaOH. SA was incorporated at concentrations of 1 %, 3 %, 5 %, 7 %, 9 %, 12 %, and 15 % relative to the weight of PPI to create PPI-DS. The succinylation process was carried out at room temperature for 2 h, maintaining a constant pH using 2 M NaOH. Finally, to obtain modified PPI, the solution was dialyzed with deionized water at 4 °C for 48 h and then freeze-dried. The freeze-dried modified PPI had a moisture content of 2.48 % ± 0.15 %.

Modified PPI was dissolved in deionized water to create a 20 mg/mL PPI-DS solution, which was stirred at 25 °C for 3 h. The 30 mL solution was then stored overnight at 4 °C in 50 mL beakers to ensure proper hydration. The PPI-DS solutions were subjected to sonication using an ultrasonic cell disintegrator (JY-IIN, NingBo Scientz Biotechnology Co. Ltd., Ningbo, China) at varying power outputs (0, 100, 300, 500 W) for 30 min. The ultrasound frequency was 20 kHz, with a pulse operated for 4 s and rested for 2 s. The solution was placed in an ice-water to ensure that the temperature was below 10 °C during ultrasonication. The samples treated at ultrasonic powers of 100, 300, and 500 W for 30 min were designated as PPI-DS-100 W, PPI-DS-300 W, and PPI-DS-500 W, respectively.

### 2.3 Degree of succinylation (DS)

Initially, 2 mL of the 20 mg/mL sample solution was combined with an equal volume of 1.5 % ninhydrin solution. The mixture was subsequently heated in a boiling water bath for 20 min and then cooled to room temperature using ice water. The reaction was stopped by adding 3.0 mL of 50 % ethanol once it cooled. A spectrophotometer (UV–Visible, SYSTRONICS, India) was used to measure absorbance at 570 nm for accurate estimation of succinylation (Pan et al., 2019). Absorbance was measured at 570 nm because this wavelength corresponds to the maximum absorption of the purple complex formed by ninhydrin and charged amino groups. The DS of the sample was calculated as follows:

(1) $$\mathrm{DS}\left(\%\right)=\frac{C_{0}-C_{1}}{C_{0}}\times 100\%$$

where the absorbances of the succinylated samples and the blank control are denoted by C_0_ and C_1_, respectively.

### 2.4 Particle size distribution and zeta potential

Particle size distributions and zeta potential were measured using the Zetasizer Nano ZS (Malvern, UK). To further analysis, the samples were thinned down to a concentration of 0.1 mg/mL using distilled water. All the measurements were conducted at 25 °C.

### 2.5 Surface hydrophobicity(H_0_)

Protein solutions at various concentrations were prepared using deionized water. Subsequently, 100 μL of 8 mM ANS solution (pH 7.0) was added to 4 mL of the sample solution, and the mixture was vortexed thoroughly to ensure homogeneity. Spectrofluorometric measurements were conducted with the excitation and emission wavelengths preset at 390 nm and 468 nm respectively, with 5 nm monochromator slit settings for both channels. The initial slope of the curve which shows the relationship between fluorescence intensity and protein concentration was used to identify the *H*_*0*_ value (Li, Liu, Liu, & Wang, 2025).

### 2.6 Protein flexibility

Protein conformational flexibility was evaluated following an adapted protocol based on Yan et al. (2021). A 4 mL aliquot of protein solution was vortexed with 250 μL trypsin solution prepared using Tris-HCl buffer (pH 8.0), followed by incubation at 37 °C for 15 min under controlled thermal conditions. Enzymatic digestion was subsequently quenched by the addition of 4 mL 10 mg/mL trichloroacetic acid. The resultant mixture underwent centrifugation at 1844 x*g* for 30 min, and the absorbance of the supernatant at λ = 280 nm was quantified using a UV–Vis spectrophotometer.

### 2.7 Protein solubility

100 mg of the protein samples were weighed and dispersed into 20 mL of deionized water to ensure complete hydration. The mixture was centrifuged at 4722 x*g* for 15 min, and the supernatant was collected. Soluble protein content determination employed the BCA colorimetric assay calibrated against bovine serum albumin standards (Zhang, Wang, et al., 2023). The formula below is used to calculate protein solubility:

(2) $$\mathrm{Solubility}\left(\%\right)=\frac{B_{1}}{B_{0}}\times 100\%$$

where B_0_ and B_1_ are defined as the total protein concentration and soluble protein concentration, respectively.

### 2.8 Fourier transform infrared spectroscopy (FTIR)

After a homogenous mass ratio of 1:100 was applied to the protein powders and potassium bromide (KBr), the mixture was pressed into transparent sheets in order to be measured using FTIR spectroscopy. The spectra were acquired with 32 scans at 4 cm^−1^ resolution, covering the wavenumber range of 4000–400 cm^−1^ (Ji et al., 2025).

### 2.9 Interfacial tension

The interfacial tension was measured by dynamic during drop method. Soy oil was placed in an oil bath, and a 10 μL drop of the solution of samples was automatically extruded from the upper line tube, and the computer automatically calculated the interfacial tension based on the shape of the drop. The change of interfacial tension was continuously monitored for 10,800 s (Hong, Kong, Chen, Guo, & Huang, 2025).

### 2.10 Emulsifying activity index (EAI) and emulsion stability index (ESI)

EAI and ESI were using the method of Peng et al. (2016). The emulsions (50 μL) were dispersed into SDS solution (5 mL, 0.1 %), and Microplate Reader was used to measure solutions (kept for 0 min and 10 min) at 500 nm. These equations were used to calculate the EAI and ESI:

(3) $$\mathrm{ESI}\left(\min\right)=\frac{A_{10}}{A_{0}}\times 100\%$$

(4) $$\mathrm{EAI}\left(m^{2}/g\right)=\frac{2.303\times 2\times A_{0}\times N}{10000\times \phi \times C\times L}\;$$

The N, ϕ, L, and C corresponded to the dilution multiple (100), soy oil volume fraction (0.25), cuvette path length (1 cm), and protein concentration (g/mL), respectively.

### 2.11 Preparation of HIPEs

The different samples of PPI were respectively dispersed in deionized water at concentrations of 1.0%. Then the dispersion was respectively mixed with soy oil in a 1:4 ratio and homogenized using a high-speed homogenizer (IKA-T25, Germany) at 10000 ×g for 2 min to obtain the HIPEs.

### 2.12 Confocal laser scanning microscopy (CLSM)

A CLSM (FV3000, Olympus, Japan) was used with 633 nm of excitation wavelength and 488 nm. Nile red and Nile blue were utilized to stain oil and protein components, respectively (Yuan, Mei, & Xie, 2024).

### 2.13 Rheological properties of HIPEs

Rheological measurements of HIPEs were conducted using an AR2000ex rheometer (TA Instruments, Crawley, UK) equipped with a 40 mm diameter parallel plate fixture with a gap set and maintained at 1 mm. The measurements of shear viscosity, three-interval thixotropy test (3ITT), creep-recovery and frequency sweep were determined by our previous method (Shen et al., 2025).

#### 2.13.1 Shear viscosity

The sample was positioned on the platform, and the shear rate was varied from 0.1 to 100 s^−1^. By measuring the response of the sample to different shear rates, apparent viscosity of HIPEs was determined.

#### 2.13.2 Three-interval thixotropy test (3ITT)

3ITT were performed on the samples by rheometer for three stages, which maintained shear rate of 1 s^−1^ in the first and third stages for a duration of 200 s and applied shear rate of 100 s^−1^ in the second stage for 200 s. All the tests were conducted at 25 °C.

#### 2.13.3 Creep-recovery

In the first phase, constant stress of 1 Pa was applied for 100 s on HIPEs to assess their stress response. The stress applied to the samples was immediately withdrawn at the beginning of the second phase to enter the recovery phase (200 s).

#### 2.13.4 Frequency sweep

The frequency sweep was conducted across the range of 0.1 to 10 Hz, maintaining a constant strain of 1 % to ensure measurements within the linear viscoelastic region. The values of G′ (storage modulus) and G″ (loss modulus) were recorded.

### 2.14 3D-printing performance of HIPEs

A food 3D printer (SHINOVE-D1, Shiyin Tech Co. Ltd., Hangzhou, China) was used to carry out the 3D printing process. The HIPEs inks were extruded at a temperature of 25 °C using a 1.2 mm nozzle. The shape printed was a hollow cross (40 mm × 40 mm × 20 mm).

### 2.15 Statistical analysis

The SPSS 11.7 software was employed for statistical analysis, with data shown as mean ± standard deviation and a significance level set at *p* < 0.05. Graphs were created using Origin 2023 software (OriginLab, USA).

## 3 Results and discussion

### 3.1 Degree of succinylation (DS)

**Fig. 1:**
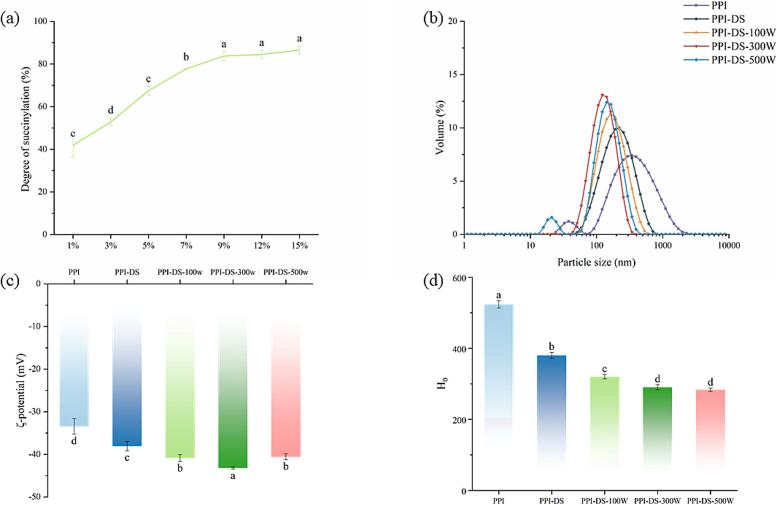
Degree of succinylation of PPI with different SA additions (a), Particle size distributions (b), Zeta potential (c) and Surface hydrophobicity (d) of PPI and PPI-DS with different ultrasound powers. a–e: Different lower letters above error bars indicate significant difference (*P* < 0.05) among different samples.

Succinylation is primarily a nucleophilic substitution reaction where the succinyl group functional moiety interacts with amino acid residues of proteins (ε-amino, hydroxyl, sulfhydryl groups) (Basak & Singhal, 2022). The degree of succinylation determines the degree to which the structure and functional properties of the protein are altered. The addition of SA to the PPI solution resulted in an increase in DS as the amount of SA increased (Fig. 1a). As the SA concentration increased from 0 to 5 %, the DS displayed a nearly linear rise to 67.51 %. When the SA concentration further increased, the rate of the DS increase slowed. This phenomenon was consistent with the finding of Lian et al. (2023). This phenomenon occurs because SA preferentially undergoes acylation reactions with lysine residees on the PPI surface, upon reaching a critical DS, and it proceeds to react with internal residues. The critical DS for PPI is about 68 %, suggesting that approximately 68 % of the lysine residues are located at or near the protein surface. Therefore, significant changes in PPI structure occur only after the acylation of these surface amino groups is completed. The DS reached a maximum value of 83.77 % at 9 % SA and plateaued at higher concentrations (>9 %). This might be due to the active sites on the proteins that could be modified gradually reaching a saturated state, thereby reducing the production of the target product (Zhao et al., 2017). Therefore, the SA concentration of 9 % was selected for subsequent experiments.

### 3.2 *Zeta* (ζ)-*potential*

ζ-potential serves as a powerful tool for analyzing proteins, offering understanding of their surface charge characteristics, conformational alterations, and stability (Ni et al., 2025). Following succinylation, the ζ-potential values of PPI decreased, reflecting an increase in negatively charged groups on the protein surface (Fig. 1c). The replacement of protonated amino groups with negatively charged succinic anhydride groups increased the net negative charge on the PPI surface, enhancing electrostatic repulsion between protein particles. A prior study by Lang et al. (2024) similarly reported a decrease in the ζ-potential of succinylated myofibrillar proteins. Subsequently, as the ultrasonic power was increased from 100 W to 300 W, the ζ-potential of PPI-DS further declined. Ultrasonication treatment might cause protein structural breakdown and chain unfolding, exposing and accumulating previously hidden charged groups along the chains, thereby exposing more negative charge residues on the protein surface to solvent molecules (Thakur et al., 2025). However, with a further rise in ultrasonic power, the ζ-potential of PPI-DS showed a slight increase, indicating that re-aggregation of internal groups in PPI. Moreover, the ζ-potential of proteins plays a crucial role in emulsion stability, as charged proteins adsorbed at interfaces can increase electrostatic repulsion between droplets, thereby minimizing droplet aggregation caused by van der Waals forces (Shi et al., 2023). The findings indicated that the hydrophilic-hydrophobic balance in protein molecular structures was effectively disrupted by succinylation, resulting in a significant impact when combined with ultrasonication treatment.

### 3.3 Particle size

Protein particle size is intrinsically linked to their functional properties, significantly influencing PPI processing quality. Proteins with smaller average particle sizes are beneficial for emulsion formation and stabilization. The native PPI exhibited a bimodal distribution (Fig. 1b). Succinylation treatment significantly altered the particle size distribution of the PPI-DS sample, shifting the main peak to smaller diameters and eliminating the secondary peak. The result was mainly due to the ongoing addition of negatively charged acyl groups, which increased the surface area of protein molecules and generated strong electrostatic repulsion, thereby preventing protein aggregation and decreasing overall particle size (Okagu, Jin, & Udenigwe, 2021). The particle size distribution of PPI moved to the left and then to the right as ultrasonic power increased, with the smallest size observed at 300 W. This phenomenon is likely due to the intense local pressures and temperatures from ultrasonic cavitation, which disrupt protein–protein interactions (such as hydrogen bonds, hydrophobic interactions, and electrostatic interactions), leading to the depolymerization of proteins into smaller particles (Sun et al., 2022). This result aligned with the findings of Sun et al. (2025), who demonstrated that ultrasonic-assisted pH shift treatment caused unfolding of soy/potato proteins, significantly reduced protein aggregate sizes and confirmed the efficacy of dual modification. However, as ultrasonic power further increased, the particle size distribution of PPI-DS-500 W showed a clear bimodal pattern, with the main peak moving towards larger diameters. Excessive ultrasonic power could cause proteins to denature too much and form small aggregates, resulting in a larger average particle size and the occurrence of secondary peak. Liu et al. (2023) also reported a similar phenomenon, who that ultrasonic waves of different powers caused varying degrees of disruption to the internal chemical bonds of proteins, leading to their unfolding and subsequent re-aggregation.

### 3.4 Surface hydrophobicity(H_0_)

The *H*_*0*_ of proteins refers to the number of hydrophobic groups distributed on the molecular surface that are capable of forming hydrophobic bonds. This property reflects the structural stability of the protein and critically influences its functional characteristics. As shown in Fig. 1d, after succinylation treatment, the *H*_*0*_ of the PPI-DS sample significantly decreased. On one hand, succinylation modification introduced hydrophilic succinamide groups, which induced conformational changes in PPI protein molecules, thus affecting the burial and folding of surface amino acids and decreasing the proportion of exposed hydrophobic moieties. On the other hand, the net negative charges introduced by succinylation enhanced intermolecular electrostatic repulsion as proven by the ζ- potential results, which sterically hindered the approach and binding of ANS fluorescent probes to hydrophobic regions on the protein surface, resulting in a measurable decline in *H*_*0*_ (Wu et al., 2022). Furthermore, the *H*_*0*_ of PPI-DS exhibited a marked reduction with increasing ultrasonic power. This phenomenon likely resulted from ultrasonic-induced mechanical shearing, turbulence, thermal effects, and dynamic stirring, which might disrupt intramolecular hydrophobic interactions within protein structures, leading to the exposure of hydrophilic functional groups through structural reorganization (Cao et al., 2021). Furthermore, although ultrasonic treatment might cause protein unfolding, it can simultaneously promote the rearrangement of hydrophobic regions within the protein molecules, leading to the re-encapsulation of previously exposed hydrophobic groups and thereby reducing *H*_*0*_ (Patil et al., 2024). This finding strongly supported that combining physical and chemical modifications significantly influenced changes in protein spatial conformation.

### 3.5 Protein flexibility

**Fig. 2:**
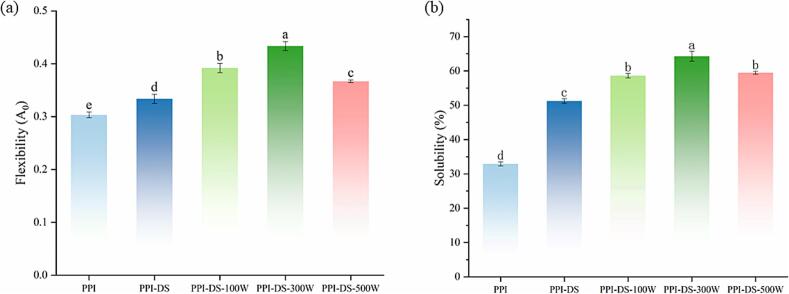
Protein flexibility (a)and Solubility (b) of PPI and PPI-DS with different ultrasound powers. a–e: Different lower letters above error bars indicate significant difference (P < 0.05) among different samples.

The protein molecular flexibility refers to the relative motion of various structural regions within a protein or the rearrangement rate of amino acid residues in the polypeptide chain, which can reflect the extensibility of the protein and plays a crucial role in its foaming and emulsifying properties (Li, Liu, Tan, Wu, & Wu, 2025). Native PPI exhibited low molecular flexibility, while its flexibility increased after succinylation modification (Fig. 2a). Unmodified proteins possessed a compact structure with low molecular flexibility, whereas succinylated PPI-DS underwent structural unfolding. The protein molecules lost their rigid tertiary structure, exposing more flexible regions and thereby enhancing molecular flexibility. With increasing ultrasonic power, the molecular flexibility of the PPI-DS exhibited an initial rise followed by a decline, peaking at an ultrasonic power of 300 W. Ultrasonication induced cavitation, leading to the formation and collapse of bubbles that disrupted weak intramolecular interactions. Concurrently, shear forces physically stretched polypeptide chains, which destabilized rigid secondary structures and promoted conformational relaxation, thus enhancing protein molecular flexibility (Liu et al., 2024; Xu et al., 2019). However, with further increases in ultrasonic power, excessive energy input caused disordered aggregation or partial protein denaturation, resulting in decreased molecular flexibility of proteins. These findings suggest that succinylation and moderate ultrasonication synergistically optimize protein flexibility by balancing structural unfolding and stability, thereby improving functional properties of the protein.

### 3.6 Protein solubility

Protein solubility is linked to its functional properties and is affected by protein denaturation and aggregation states. Compared to native PPI (32.93 %), the solubility of PPI-DS gradually increased to 51.23 % (Fig. 2b). The introduction of hydrophilic succinyl groups with negative charges into PPI boosted electrostatic repulsion among protein molecules and disrupted their interactions. This resulted in a looser protein structure which increased the contact area between the protein surface and water molecules. Simultaneously, the exposed hydrophilic groups within the molecule enhanced protein hydration. These effects collectively increased the solubility of PPI (Charoensuk, Brannan, Chanasattru, & Chaiyasit, 2018). As the ultrasonic power increased, the solubility of PPI-DS exhibited an initial increase followed by a slight decrease. The intense mechanical forces from ultrasound led to protein unfolding and peptide bond breakage, enhancing the protein-water interactions (Mir, Riar, & Singh, 2019). The reduction in average particle size due to ultrasonic treatment could be another factor contributing to the rise in protein solubility, as smaller particles have a greater contact area with water molecules.

### 3.7 Ft-IR

**Fig. 3:**
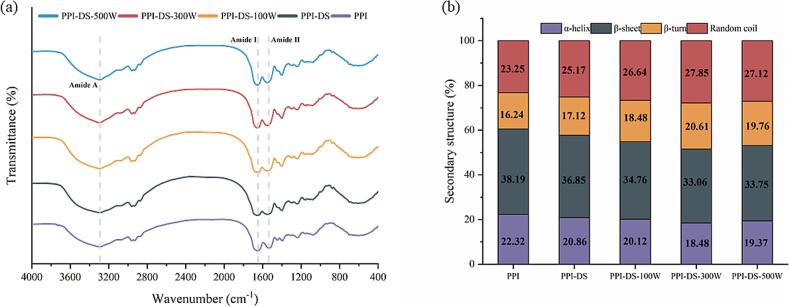
FT-IR spectra (a) and Secondary structure content (b) of PPI and PPI-DS with different ultrasound powers.

As shown in Fig. 3a, the amide A band (3200–3400 cm^−1^) corresponds to N—H and O—H stretching vibrations, while the amide I (1600–1700 cm^−1^) and amide II (1480–1580 cm^−1^) regions are associated with C=O and C—N stretching, and N—H and C—N bending vibrations, respectively (Chen et al., 2025). Succinylation of PPI resulted in slight redshifts at amide I and amide II. In addition, the peaks at amide A band for PPI-DS become progressively narrower. This was attributed to the reaction between free amino groups and succinyl groups, which converted N—H bonds to C—N bonds.The characteristic peaks at 1397 cm^−1^ corresponding to COO- stretching vibrations, exhibited redshifts and enhancement compared to PPI, indicating that succinylation increased the -COOH groups content (Hu et al., 2022).These findings suggested that succinyl groups formed covalent bonds with protein amino groups (-NH₂), generating succinylated products. The amide A peak position of PPI-DS was redshifted as the ultrasonic power increased, which was due to ultrasound disrupting non-covalent interactions involving N—H or O—H, leading to PPI dissociation. There was a slight shift of the amide I and amide II peak, suggesting that in addition to succinylation, ultrasound caused further alterations in the protein secondary structure (Khatkar, Kaur, Khatkar, & Mehta, 2018).

FT-IR spectroscopy was used to determine the secondary structure of both natural PPI samples and ultrasound-treated PPI-DS samples. Fig. 3b illustrates that native PPI contains 38.19 % β-sheet, 22.32 % α-helix, 16.24 % β-turn and 23.25 % random coil. Compared with native PPI, succinylation treatment led to a gradual reduction in the α-helix (20.86 %) and β-sheet (36.85 %) content within the ordered structure of PPI-DS samples. The content of β-turn (17.12 %) and random coil (25.17 %) in its disordered structure increased. This might be due to succinylation-induced unfolding of protein molecules, which facilitated the transition from ordered spatial conformations to disordered random structures, thereby enhancing conformational flexibility (Wan et al., 2018). These results align with the observed increase in protein flexibility. Moreover, ultrasonication further disrupted the ordered protein structure, leading to a continuous decrease in α-helix (18.48 %) and β-sheet (33.06 %) content of PPI-DS-300 W, with a greater loss observed in β-sheet. This occurred because α-helix structures have tightly packed conformation and enhanced stability, while β-sheet are more vulnerable to hydrogen bond breakage owing to their less stable architecture. Other researchers reported that the decreases in α-helix structures and increases in random coils facilitated protein-protein interactions, thereby enhancing the stability of protein films formed with oil droplets (Liu, Zhang, Wang, Chen, & Kong, 2021). In summary, dual modification of PPI led to a more disordered secondary structure and such flexible protein moleculars could be expected to show better emulsifying properties.

### 3.8 Interfacial tension analysis

**Fig. 4:**
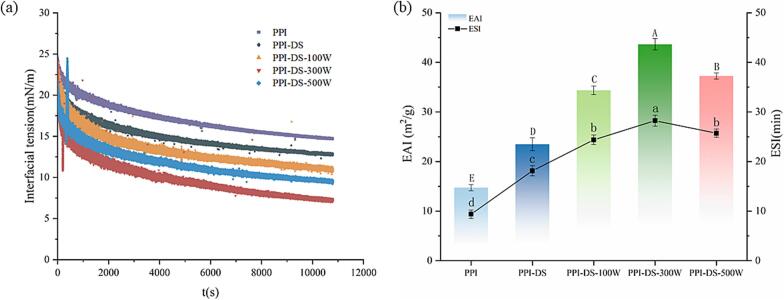
Interfacial tension (a) and Emulsifying properties (b) of PPI and PPI-DS with different ultrasound powers. a–e: Different lower letters above error bars indicate significant difference (P < 0.05) among different samples.

Proteins are capable of stabilizing emulsion systems by decreasing the interfacial tension between oil and water. Typically, reduced dynamic interfacial tension indicates enhanced adsorption at the oil-water interface (Huang, Luo, Ning, Ye, & Liu, 2024). The adsorption at the oil-water interface mainly occurs in two stages (Fig. 4a). In the first stage, as the oil-water interfacial tension rapidly decreases, as PPI molecules adsorb at the oil-water interface. During the second stage, adsorbed PPI molecules rearrange to form a viscoelastic film, gradually reducing interfacial tension until dynamic equilibrium is achieved. The succinylated PPI decreased the oil-water interfacial tension, indicating that the modification balanced the hydrophilic/hydrophobic surfaces of protein, enhancing interfacial adsorption. The interfacial tension decreased with increasing ultrasonic power (0–300 W), suggesting that optimal ultrasonication enhances the adsorption efficiency of PPI-DS at the oil-water interface, thereby reducing interfacial tension. This outcome was attributed to the capacity of ultrasound treatment to directly interact with protein molecules, improving their flexibility and surface properties, which likely facilitates rapid rearrangement at the oil-water interface and enhances interfacial activity (Hong et al., 2024). Tang (2017) also found that protein denaturation with high flexibility could enhance their surface properties. However, the higher ultrasonic power (500 W) caused PPI-DS aggregation and reduced solubility, thus inhibiting the protein adsorption to some degree.

### 3.9 Emulsifying properties

The emulsifying properties of proteins represent their ability to adsorb at oil-water interfaces. The EAI of PPI-DS was higher than native PPI (Fig. 4b). The EAI of PPI-DS first increased and then decreased as ultrasonic power increased. The ESI of PPI exhibited a similar trend with EAI. The decrease in particle size and the rise in electronegativity and solubility enhanced protein molecule migration to the oil-water interface, thereby improving emulsifying properties of protein (Zhao, Hong, Fan, Liu, & Li, 2022). In addition, the introduction of carboxyl groups via acylation enhanced interactions between protein molecules and the aqueous phase. Simultaneously, dual succinylation and ultrasonication modification induced further structural unfolding and rearrangement of the protein, strengthening interactions at the protein-oil interface. Following adsorption at the oil-water interface, flexible proteins underwent relatively rapid conformational changes, further optimizing their emulsifying performance. However, high-intensity ultrasonication might disrupt the charged groups introduced by chemical modification, thereby weakening electrostatic stabilization. This could induce partial protein aggregation, leading to an increase in interfacial tension, which subsequently inhibited the adsorption and accumulation of protein molecules at the interface (Ma et al., 2019). Consequently, the droplets tended to aggregate, leading to lower EAI and ESI. Our findings aligned with those of Patil et al. (2024).

### 3.10 Microstructure of HIPEs

**Fig. 5:**
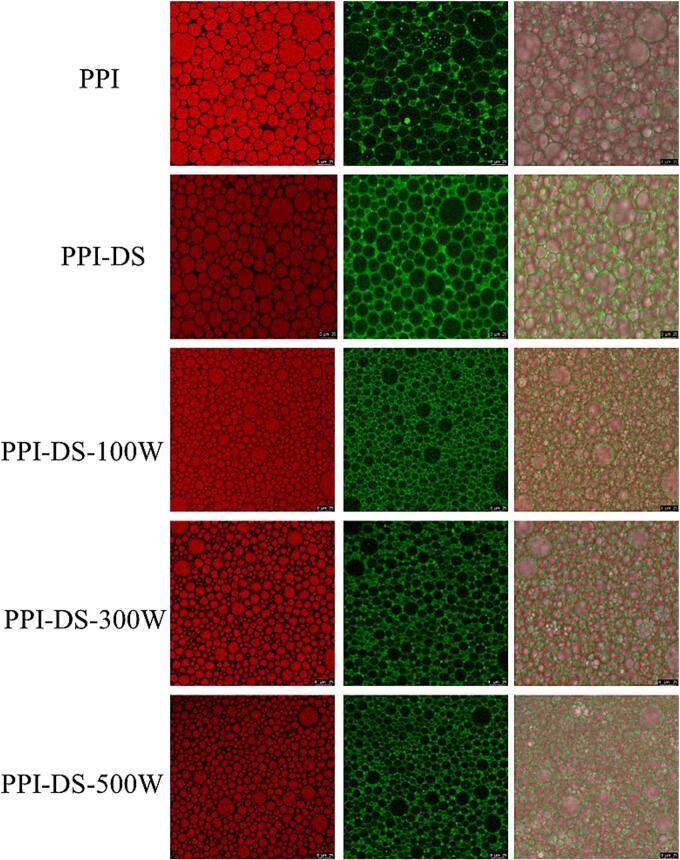
CLSM of HIPEs stabilized by PPI and PPI-DS with different ultrasound powers. PPI was stained with Nile blue (blue), and soy oil was stained with Nile red (red). (For interpretation of the references to colour in this figure legend, the reader is referred to the web version of this article.)

The oil-water interfacial microstructure and droplet distribution of HIPEs were characterized using CLSM. Fig. 5 shows the microstructure of HIPEs stabilized by PPI alone, PPI-DS and PPI-DS modified with varying ultrasonic power. Green and red fluorescence represent PPI (water phase) and soybean oil (oil phase), respectively. The green fluorescence around the droplets indicated the adsorption of PPI at the oil-water interface. Compared to PPI, the droplets size in PPI-DS slightly decreased. The introduction of succinyl group modified the surface charge of emulsion, increasing electrostatic repulsion between droplets and thereby enhancing stability (Shilpashree, Arora, Chawla, & Tomar, 2015). Additionally, the ultrasonication led to more uniform size of emulsion droplets. The red oil droplets stabilized by PPI-DS-300 W appeared smaller and more homogeneous in distribution. The enhancement was ascribed to ultrasonication, which altered protein conformation by disrupting disulfide bonds and non-covalent interactions. These increased protein flexibility and decreased interfacial tension, which enhanced protein's adsorption rate and coverage density at the oil-water interface, thereby stabilizing smaller droplets (Diao et al., 2024). However, the occurrence of partial large droplets in PPI emulsions at high intensity ultrasound might be caused by the low solubility of PPI, resulting in reduced charge density and consequently weaker electrostatic repulsion.

### 3.11 Rheological properties of HIPEs

The 3D printing process could be divided into three stages: the extrusion stage, the recovery stage, and the self-supporting stage. Each stage could be characterized by distinct rheological parameters of HIPEs. The flow behavior of HIPEs during 3D printing was critical for their ability to pass through the nozzle, uniformly coat the substrate, and regain viscosity post-printing to maintain high-resolution shapes. Rheological analysis was essential for evaluating the self-supporting properties and printability of edible inks, contributing to innovative food design. To predict the printability of HIPEs and reduce the number of additional printing trials, tests and analyses were conducted on the extrudability (shear rate tests), recoverability (3ITT and creep recovery tests), and self-supporting properties (frequency sweep tests) of HIPEs.

#### 3.11.1 Apparent viscosity

**Fig. 6:**
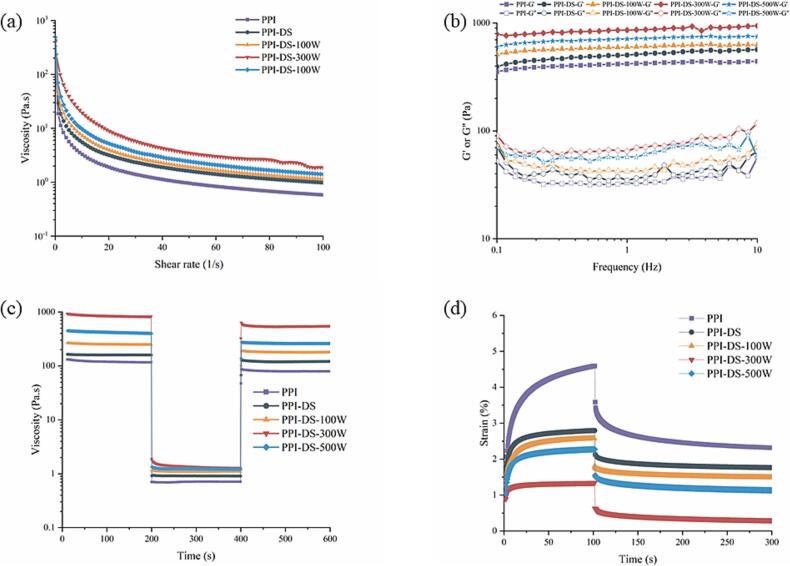
Shear rate sweep (a), frequency sweep (b), 3ITT (c) and Creep recovery (d) of HIPEs based on HIPEs constructed by PPI and PPI-DS with different ultrasound powers.

Viscosity is a critical indicator reflecting the feasibility of 3D printing with HIPEs. During the 3D printing process, optimal ink viscosity not only ensures robust interlayer adhesion and enhanced structural integrity but also mitigates the risk of delamination or collapse in printed objects. In the extrusion stage, HIPEs must exhibit shear-thinning behavior to enable smooth passage through the narrow nozzle of the printer. As shown in Fig. 6a, the apparent viscosity of HIPEs showed a negative correlation with shear rate, which was canonical of shear thinning behavior of non-Newtonian fluids, facilitating seamless extrusion from the 3D printer nozzle (Shang et al., 2023). Furthermore, compared to PPI-stabilized HIPEs, those stabilized by PPI-DS exhibited increased apparent viscosity. This might result from smaller emulsion droplets formed by PPI-DS, which packed more densely, thereby strengthening molecular interactions between oil droplets and the continuous phase to create a tighter gel network (Ji, Wang, et al., 2025). Consequently, droplet mobility was reduced, leading to higher apparent viscosity. Notably, the apparent viscosity of HIPEs further rose with the ultrasonic power increasing from 100 W to 300 W. Appropriate ultrasonic treatment promoted the unfolding of PPI molecules, thus exposing more charged groups. This enhanced the adsorption of PPI molecules at the oil-water interface, leading to the formation of denser and more stable interfacial films. The reinforced interfacial films could inhibit droplets coalescence, which increased flow resistance in the continuous phase and elevated viscosity (Xu et al., 2025). Stokes' law indicates that higher viscosity decreases droplet sedimentation and flotation rates, thereby improving emulsion stability. However, the viscosity of HIPEs stabilized by PPI-DS-500 slightly decreased. High-power ultrasonic pretreatment altered the protein structure, decreasing protein flexibility and resistance to flow between emulsion droplets, which led to reduced viscosity. An et al. (2025) reported similar results that increased ultrasonic power likely caused protein aggregation, reducing the resistance to fluid flow between droplets.

#### 3.11.2 3ITT

The 3ITT simulated various shear rates encountered before, during, and after 3D printing of HIPEs to assess their printability. The 3ITT results are depicted in Fig. 6c. Initially, the HIPEs exhibited high viscosity, which remained stable over time. Secondly, increasing the shear rate to 100 s^−1^ led to a rapid decrease in the viscosity of the HIPEs. At high shear rates, viscosity loss was more pronounced. Finally, reducing the shear rate to 0.1 s^−1^ led to a rapid increase in the viscosity of the HIPEs, demonstrating the swift recovery of the emulsion's network structure upon stress removal, a critical factor for 3D printing (Ji, Gao, et al., 2025). In all PPI samples, viscosity was lower in the third stage compared to the initial stage. This might be because the non-covalent interactions in HIPEs were disrupted by shear and did not fully recover. Additionally, the viscosity recovery ability of HIPEs with different samples was significantly different. Specifically, the viscosity recovery ability of PPI-DS was higher than that of PPI. In addition, the viscosity recovery of HIPEs improved with increasing ultrasonic power, and the HIPEs stabilized by PPI-DS-300 W had the best recovery performance. The higher recovery performance enables rapid restoration of structural and mechanical properties, thereby facilitating adequate support for the subsequent extrusion layer. Succinylation combined with ultrasonication allowed PPI molecules to arrange densely and orderly at the oil-water interface, strengthening the network structure and maximizing resistance to instant deformation. The recovery performance of the HIPEs notably declined at 500 W. High-power ultrasound caused the hydrophobic and charged groups on the protein surface at the interface to become buried, weakening steric hindrance and electrostatic repulsion between emulsion droplets, thereby impeding the formation of a strong gel network and facilitating a weaker one.

#### 3.11.3 Creep recovery tests

Creep recovery is defined as the phenomenon where a solid material exhibits increasing strain under constant stress over time, followed by a gradual reduction in strain upon stress removal. Specifically, the application of stress mimics the shear forces experienced by HIPEs during extrusion through a 3D printer nozzle, while the recovery phase corresponds to the structural stabilization of printed layers after extrusion (Li, Wang, Yang, Lv, & Xiao, 2025). HIPEs with favorable creep recovery properties can effectively maintain spatial integrity during 3D printing, particularly advantageous for fabricating intricate architectures. As shown in Fig. 6d, a constant stress of 1 Pa was applied from 1 to 100 s, followed by stress removal from 100 to 300 s in the experiment. Under constant stress, all HIPEs exhibited rapid strain increase, which subsequently decreased after stress removal. Generally, creep recovery compliance reflects the softness of the system, where higher values indicate weaker structural integrity (Shen et al., 2025). Compared with PPI, the HIPEs stabilized by PPI-DS exhibited a significantly lower maximum creep value, indicating that succinylation modification of PPI improved the deformation resistance of HIPEs. The succinylation modification improved the interfacial adsorption properties of PPI, contributing to the formation of a more stable network structure at the droplet surface, thereby increasing the resistance of HIPEs to deformation. Consequently, this enhanced deformation resistance enables better tolerance to extrusion shear forces during 3D printing, resulting in final products with higher dimensional resolution. The extent of non-recoverable deformation after stress removal, stemming from irreversible viscous flow strain during creep, varied between samples. Notably, under the same succinylation conditions, the reinforcement effect of PPI-DS-300 W was more pronounced, suggesting the formation of a highly cross-linked network structure in HIPEs with less structural disintegration and irreversible damage. This could be attributed to increased protein-protein interactions as protein particle size decreases, leading to a more compact gel network that improves the creep recovery capability of HIPEs.

#### 3.11.4 Frequency scan tests

During the self-supporting stage, HIPEs with sufficient G′ could ensure shape fidelity after printing. The G' of all HIPEs exceeded G" across the entire frequency range (Fig. 6b), demonstrating their predominantly gel-like elastic behavior. Besides, the G′ and G" values of the HIPEs exhibited minimal frequency dependence, suggesting that the HIPEs structures are stable against frequency variations (Xie et al., 2025). HIPEs stabilized by PPI-DS exhibited higher G' and G“ values. This suggested their enhanced mechanical strength, potentially improving printability. This might be due to succinylation promoting a more flexible conformation, which formed a denser protective film at the interface and encapsulated smaller droplets. This enhanced the cross-linking of interfacial proteins and droplet interactions at the oil-water interface, leading to a stronger network structure. The G′ and G" values of the PPI-DS- HIPEs initially rose and then fell with increasing ultrasonic power, reaching the maximum value at 300 W. Appropriate ultrasonic treatment might disrupt protein aggregates and covalent bonds in peptide chains, enhancing protein molecular mobility and facilitating transfer to the oil–water interface, thereby improving emulsion viscoelasticity (Hong et al., 2024). Therefore, the HIPEs exhibited sufficient elasticity to maintain self-support, an essential for 3D-printing food inks to preserve shape fidelity post-printing. Increasing ultrasonic power further led to a decrease in the G′ and G″ of the HIPEs. Ultrasonic thermal effects might lead to protein aggregation, which fills the interfacial layers between adjacent droplets and thereby weakens the viscoelasticity of network structure. In conclusion, dual succinylation and ultrasonication modifications positively influenced the properties of HIPEs through conformational changes in the protein.

### 3.12 3D-printing performance of HIPEs

**Fig. 7:**
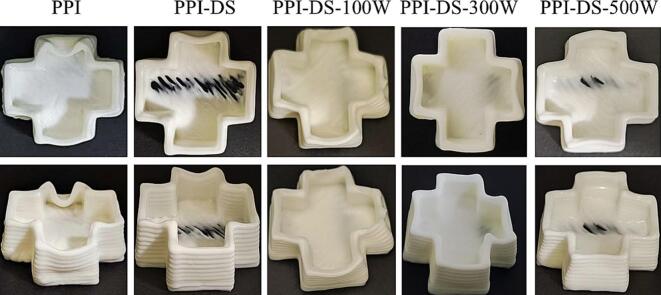
3D printability of HIPEs stabilized by PPI and PPI-DS with different ultrasound powers.

3D printing of food materials offers a novel processing paradigm for the food industry, enabling the creation of appealing textured patterns. Generally speaking, high-quality printed products are typically characterized by structural stability, smooth surfaces, and precise detailing (Zhang et al., 2024). To evaluate the printability of HIPEs, a “cross” shape was printed (Fig. 7). While natural PPI-stabilized HIPEs could form three-dimensional structures, the printed objects exhibited rough surface textures and poor shape fidelity. This might stem from the low mechanical strength of the HIPEs, resulting in insufficient self-supporting capacity and compromised surface integrity of the printed samples. In contrast, PPI-DS-stabilized HIPEs displayed well-defined “cross” shapes with enhanced structural resolution and shape fidelity, suggesting that succinylation modification improves the printing stability of HIPEs. As ultrasonic power increased, the HIPEs demonstrated higher printing resolution, improved self-supporting structures, and a more visually appealing appearance. This improvement might arise from ultrasonication further increasing the surface charge of PPI-DS, promoting fuller adsorption of protein molecules at the oil-water interface. Consequently, more charged groups were incorporated into the interfacial film, strengthening electrostatic repulsion between droplets. The dual succinylation and ultrasonication modification allowed PPI to form a unique networked structure at the oil-water interface, thereby enhancing the mechanical strength of HIPEs. Furthermore, the improved G′ and shear recovery viscosity contributed to the superior printability of HIPEs. However, when the ultrasonic power was further increased to 500 W, significant layer fusion occurred in the printed samples. This likely resulted from insufficient viscosity and mechanical strength, causing structural collapse during high-speed shear processes and delayed recovery. High-intensity ultrasound weakened the interfacial adsorption of PPI-DS, leading to the formation of weak interfacial layers. These weak layers failed to provide adequate deformation resistance for oil droplets, allowing droplet aggregation or rearrangement during 3D printing and ultimately destabilizing the printed structure.

## 4 Conclusion

This study investigated the impact of dual modification via succinylation and ultrasonication on the physicochemical properties of PPI, along with the rheological properties and 3D printing capabilities of PPI-HIPEs. Ultrasonic cavitation and succinylation changed the PPI structure, resulting in more flexible protein conformation. This promoted protein adsorption at the oil-water interface and decreased interfacial tension. These changes were crucial for stabilizing HIPEs. Dual succinylation and ultrasonication treatments promote the unfolding and cross-linking of interfacial PPI, leading to the formation of a dense film that covers more oil-water interface. This not only improved the network structure of HIPEs, but also boosted their apparent viscosity, mechanical strength and recovery characteristicsof HIPEs in 3D printing. In contrast, high-intensity ultrasound induced protein aggregation, destroying the interfacial adsorption layer, thus weakening properties of HIPEs. This study provides an elucidation of the mechanism underlying the conformational changes in succinylated PPI induced by ultrasound treatment, as well as the formation of PPI-HIPEs.

## CRediT authorship contribution statement

**Fuwei Sun:** Writing – original draft, Software, Conceptualization. **Genna Ba:** Data curation. **Chaojiang Dong:** Visualization. **Zhijun Fan:** Methodology. **Hongjian Zhang:** Investigation. **Lulu Shen:** Investigation. **Lianzhou Jiang:** Investigation. **Zhongjiang Wang:** Writing – original draft, Supervision, Conceptualization. **Zengwang Guo:** Project administration, Funding acquisition.

## Funding

This research was supported by the National key R&D plan in the 14th five year plan (2021YFD2100401), Harbin Manufacturing Scientific and Technological Innovation Talent Program (2022HBRCCG005), and the 10.13039/5011000085342023 youth leading talent support plan of Northeast Agricultural University (NEAU2023QNLJ-007).

## Declaration of competing interest

The authors declare that they have no known competing financial interests or personal relationships that could have appeared to influence the work reported in this paper.

## Data Availability

Data will be made available on request.

## References

[bb0005] An Y., Guo R., Gao Y., Zhu Y., Huang Y., Liu L., Zhu X. (2025). Ultrasonic treatment of emulsion gels with different soy protein-hemp protein composite ratios: Changes in structural and physicochemical properties. International Journal of Biological Macromolecules.

[bb0010] Basak S., Singhal R.S. (2022). Succinylation of food proteins-a concise review. Lwt.

[bb0015] Cao H., Sun R., Shi J., Li M., Guan X., Liu J., Zhang Y. (2021). Effect of ultrasonic on the structure and quality characteristics of quinoa protein oxidation aggregates. Ultrasonics Sonochemistry.

[bb0020] Charoensuk D., Brannan R.G., Chanasattru W., Chaiyasit W. (2018). Physicochemical and emulsifying properties of mung bean protein isolate as influenced by succinylation. International Journal of Food Properties.

[bb0025] Chen H., Fei X., Liu R., Zhang J., Gong D., Hu X., Zhang G. (2025). A combination of microwave treatment and covalent binding of gallic acid improves the solubility, emulsification and thermal stability of peanut protein isolate. Food Research International.

[bb0030] Cui Q., Zhang A., Li R., Wang X., Sun L., Jiang L. (2020). Ultrasonic treatment affects emulsifying properties and molecular flexibility of soybean protein isolate-glucose conjugates. Food Bioscience.

[bb0035] Dai C., Han S., Ma C., McClements D.J., Xu D., Chen S., Liu F. (2024). High internal phase emulsions stabilized by pea protein isolate-EGCG-Fe3+ complexes: Encapsulation of β-carotene. Food Hydrocolloids.

[bb0040] Diao X., Wang Y., Jia R., Chen X., Liu G., Liu D., Guan H. (2024). Influences of ultrasonic treatment on the physicochemical properties and microstructure of diacylglycerol-loaded emulsion stabilized with soybean protein isolate and sodium alginate. Ultrasonics Sonochemistry.

[bb0045] He X., Lu Q. (2024). A review of high internal phase Pickering emulsions: Stabilization, rheology, and 3D printing application. Advances in Colloid and Interface Science.

[bb0050] Hong Z., Kong Y., Chen J., Guo R., Huang Q. (2025). Collaborative stabilizing effect of trehalose and myofibrillar protein on high internal phase emulsions: Improved freeze-thaw stability and 3D printability. Food Chemistry.

[bb0055] Hong Z., Kong Y., Guo R., Huang Q. (2024). Stabilizing effect of silver carp myofibrillar protein modified by high intensity ultrasound on high internal phase emulsions: Protein denaturation, interfacial adsorption and reconfiguration. International Journal of Biological Macromolecules.

[bb0060] Hu G., Zhang J., Wang Q., Ma M., Ma L., Li S. (2022). Succinylation modified ovalbumin: Structural, interfacial, and functional properties. Foods.

[bb0065] Huang A., Luo S., Ning F., Ye J., Liu C. (2024). Preparation of protein-polyphenol-polysaccharide ternary complexes to regulate the interfacial structure of emulsions: Interfacial behavior and emulsion stability. Food Hydrocolloids.

[bb0070] Ji C., Wang Y., Ma A.W., Liang Y., Luo Y. (2025). Physiochemical and rheological characterization of plant-based proteins, pectin, and chitin nanofibers for developing Pickering emulsions as potential fat alternatives. Food Chemistry.

[bb0075] Ji Y., Gao S., Li M., Xu Y., Sun C., Hou H., Wang W. (2025). Development of high internal phase Pickering emulsions utilizing ternary complexes of octenyl succinate millet starch/chitosan hydrochloride-EGCG particles and their applications in 3D printing. Food Chemistry.

[bb0080] Kamani M.H., Semwal J., Khaneghah A.M. (2022). Functional modification of grain proteins by dual approaches: Current progress, challenges, and future perspectives. Colloids and Surfaces B: Biointerfaces.

[bb0085] Khatkar A.B., Kaur A., Khatkar S.K., Mehta N. (2018). Characterization of heat-stable whey protein: Impact of ultrasound on rheological, thermal, structural and morphological properties. Ultrasonics Sonochemistry.

[bb0090] Lang Y., Huang L., Han D., Li C., Bian P., **e, P., & Yang, X. (2024). Octenyl succinylation of myofibrillar protein: Structural, physicochemical and emulsifying properties. LWT.

[bb0095] Li G., Wang B., Yang L., Lv W., Xiao H. (2025). Effect of salt valence and ionic strength on the rheology and 3D printing performance of walnut protein emulsion gels. Food Hydrocolloids.

[bb0100] Li H., Liu Y., Tan H., Wu X., Wu W. (2025). Effect of ultrasonic pretreatment on the emulsion rheological properties and interface protein structure of epigallocatechin-3-gallate and rice bran protein complex. Food Chemistry.

[bb0105] Li S., Liu Y., Liu C., Wang C. (2025). Impact of dynamic high-pressure microfluidization on conformation and gel properties of peanut protein isolates. Journal of Food Engineering.

[bb0110] Lian Z., Yang S., Cheng L., Liao P., Dai S., Tong X., Jiang L. (2023). Emulsifying properties and oil–water interface properties of succinylated soy protein isolate: Affected by conformational flexibility of the interfacial protein. Food Hydrocolloids.

[bb0115] Liu H., Zhang J., Wang H., Chen Q., Kong B. (2021). High-intensity ultrasound improves the physical stability of myofibrillar protein emulsion at low ionic strength by destroying and suppressing myosin molecular assembly. Ultrasonics Sonochemistry.

[bb0120] Liu Q., Liu Y., Huang H., Xiong M., Yang Y., Lin C., Yang F., Xie Y., Yuan Y. (2023). Improvement of the emulsifying properties of Zanthoxylum seed protein by ultrasonic modification. Ultrasonics Sonochemistry.

[bb0125] Liu Y., Li H., Wei X., Zhou X., Wu W., Wu X. (2024). Effect of ultrasonic pretreatment on the structure and emulsion stability of epigallocatechin-3-gallate and rice bran protein complex. Lwt.

[bb0130] Ma W., Wang J., Xu X., Qin L., Wu C., Du M. (2019). Ultrasound treatment improved the physicochemical characteristics of cod protein and enhanced the stability of oil-in-water emulsion. Food Research International.

[bb0135] Mir N.A., Riar C.S., Singh S. (2019). Physicochemical, molecular and thermal properties of high-intensity ultrasound (HIUS) treated protein isolates from album (Chenopodium album) seed. Food Hydrocolloids.

[bb0140] Ni Y., Yan T., Fu K., Xu C., Zhang L., Liu D., Wang W. (2025). Enhancement of physicochemical and techno-functional properties of soy protein isolate amyloid fibrils by moderate ultrasonic pretreatment. Ultrasonics Sonochemistry.

[bb0145] Okagu O.D., Jin J., Udenigwe C.C. (2021). Impact of succinylation on pea protein-curcumin interaction, polyelectrolyte complexation with chitosan, and gastrointestinal release of curcumin in loaded-biopolymer nano-complexes. Journal of Molecular Liquids.

[bb0150] Pan Y., Xie Q.T., Zhu J., Li X.M., Meng R., Zhang B., Jin Z.Y. (2019). Study on the fabrication and in vitro digestion behavior of curcumin-loaded emulsions stabilized by succinylated whey protein hydrolysates. Food Chemistry.

[bb0155] Patil N.D., Bains A., Kaur S., Yadav R., Ali N., Patil S., Chawla P. (2024). Influence of dual succinylation and ultrasonication modification on the amino acid content, structural and functional properties of chickpea (Cicer arietinum L.) protein concentrate. Food Chemistry.

[bb0160] Peng W., Kong X., Chen Y., Zhang C., Yang Y., Hua Y. (2016). Effects of heat treatment on the emulsifying properties of pea proteins. Food Hydrocolloids.

[bb0165] Shang W., Sun Y., Song J., Zhang P., Hou Y., Wang H., Tan M. (2023). Novel high internal phase oleogels-in-water Pickering emulsions stabilized solely by whey protein isolate for 3D printing and fucoxanthin delivery. Food Hydrocolloids.

[bb0170] Shen L., Sun F., Zhang S., Lou F., Tang X., Jiang L., Guo Z. (2025). High internal phase emulsions stabilized by soy protein isolate-carboxymethyl cellulose sodium complexes: Application for 3D food printing and enhanced bioaccessibility of β-carotene. Food Hydrocolloids.

[bb0175] Shi L.S., Yang X.Y., Gong T., Hu C.Y., Shen Y.H., Meng Y.H. (2023). Ultrasonic treatment improves physical and oxidative stabilities of walnut protein isolate-based emulsion by changing protein structure. LWT.

[bb0180] Shilpashree B.G., Arora S., Chawla P., Tomar S.K. (2015). Effect of succinylation on physicochemical and functional properties of milk protein concentrate. Food Research International.

[bb0185] Sun Y., Wang L., Wang H., Zhou B., Jiang L., Zhu X. (2025). Effect of pH-shifting and ultrasound on soy/potato protein structure and gelation. Food Hydrocolloids.

[bb0190] Sun Y., Zhong M., Zhao X., Song H., Wang Q., Qi B., Jiang L. (2022). Structural and interfacial characteristics of ultrasonicated lipophilic-protein-stabilized high internal phase Pickering emulsions. Lwt.

[bb0195] Tang C.H. (2017). Emulsifying properties of soy proteins: A critical review with emphasis on the role of conformational flexibility. Critical Reviews in Food Science and Nutrition.

[bb0200] Thakur K., Sharma R., Khatkar S.K., Bobade H., Singh B., Sharma S. (2025). Isolation and ultrasonication of pearl millet protein: Effect on techno-functional, structural, molecular interaction, and rheological properties. Food Research International.

[bb0205] Wan Y., Liu J., Guo S. (2018). Effects of succinylation on the structure and thermal aggregation of soy protein isolate. Food Chemistry.

[bb0210] Wang R., Wang J.J., Guo X., Li Y., Wu Y., Liu H., Zhao Y. (2022). Physicochemical and functional properties of the Antarctic krill proteins modified by succinylation. Lwt.

[bb0215] Wang Y., Tao L., Wang Z., Wang Y., Lin X., Dai J., Tian Y. (2024). Effect of succinylation-assisted glycosylation on the structural characteristics, emulsifying, and gel properties of walnut glutenin. Food Chemistry.

[bb0220] Wu X., Gao T., Xu Z., Liu C., Teng F., Li Y. (2024). Effect of combined enzyme and ultrasound treatment on the structure and gel properties of soy protein isolate: A comparative study of alkaline protease and pepsin. Colloids and Surfaces A: Physicochemical and Engineering Aspects.

[bb0225] Wu Y., Wu Y., Xiang H., Chen S., Zhao Y., Cai Q., Wang Y. (2024). Emulsification properties and oil-water interface properties of l-lysine-assisted ultrasonic treatment in sea bass myofibrillar proteins: Influenced by the conformation of interfacial proteins. Food Hydrocolloids.

[bb0230] Wu Y., Xiang X., Liu L., An F., Geng F., Huang Q., Wei S. (2022). Ultrasound-assisted succinylation comprehensively improved functional properties of egg white protein. Lwt.

[bb0235] Xie R., Zhang S., Liu S., Gu Y., Zhang J., Guo Y., Wang Z. (2025). Stabilization of oil-in-water high internal phase Pickering emulsion by using 7S globulin/konjac glucomannan complexes: Rheology properties, stability properties and 3D/4D printing performance. Food Research International.

[bb0240] Xu H., Chandrapala J., Dabbour M., Mintah B.K., Huang L., Dai C., He R. (2025). Effect of xylose glycation and ultrasonication on the interfacial properties and physicochemical stability of silkworm pupa protein-stabilized Pickering emulsion and its applicability in emulsion-filled hydrogels. Food Research International.

[bb0245] Xu H., Zhang T., Lu Y., Lin X., Hu X., Liu L., Wu X. (2019). Effect of chlorogenic acid covalent conjugation on the allergenicity, digestibility and functional properties of whey protein. Food Chemistry.

[bb0250] Yan S., Xu J., Zhang S., Li Y. (2021). Effects of flexibility and surface hydrophobicity on emulsifying properties: Ultrasound-treated soybean protein isolate. Lwt.

[bb0255] Yuan M., Mei J., Xie J. (2024). Research Progress on polysaccharide composite films and coatings with antioxidant and antibacterial ingredients to extend the shelf life of animal-derived meat. Coatings.

[bb0260] Zhang D., Yang Y., Li R., Rong X., Zhang W., Zhang M., Zhang X. (2024). Effects of co-assembly of gliadin and carboxymethyl cellulose on the high internal phase Pickering emulsions: Rheology properties, 3D printing performance and oil-soluble nutrient delivery. Food Hydrocolloids.

[bb0265] Zhang R.Y., Wang Y., Jiang Y., Min E.H., Rao S.Q. (2023). Effects of dual succinylation and ultrasonication modification on the structural and functional properties of ovalbumin. Food Research International.

[bb0270] Zhang X., Xie W., Liang Q., Jiang X., Zhang Z., Shi W. (2023). High inner phase emulsion of fish oil stabilized with rutin-grass carp (Ctenopharyngodon idella) myofibrillar protein: Application as a fat substitute in surimi gel. Food Hydrocolloids.

[bb0275] Zhao C.B., Zhang H., Xu X.Y., Cao Y., Zheng M.Z., Liu J.S., Wu F. (2017). Effect of acetylation and succinylation on physicochemical properties and structural characteristics of oat protein isolate. Process Biochemistry.

[bb0280] Zhao Q., Hong X., Fan L., Liu Y., Li J. (2022). Solubility and emulsifying properties of perilla protein isolate: Improvement by phosphorylation in the presence of sodium tripolyphosphate and sodium trimetaphosphate. Food Chemistry.

[bb0285] Zhu Y., Fu S., Wu C., Qi B., Teng F., Wang Z., Jiang L. (2020). The investigation of protein flexibility of various soybean cultivars in relation to physicochemical and conformational properties. Food Hydrocolloids.

